# Achieving ideal transistor characteristics in conjugated polymer semiconductors

**DOI:** 10.1126/sciadv.adg8659

**Published:** 2023-06-02

**Authors:** Mingfei Xiao, Xinglong Ren, Kangyu Ji, Sein Chung, Xiaoyu Shi, Jie Han, Zefan Yao, Xudong Tao, Szymon J. Zelewski, Mark Nikolka, Youcheng Zhang, Zhilong Zhang, Zichen Wang, Nathan Jay, Ian Jacobs, Weijing Wu, Han Yu, Yarjan Abdul Samad, Samuel D. Stranks, Boseok Kang, Kilwon Cho, Jin Xie, He Yan, Shangshang Chen, Henning Sirringhaus

**Affiliations:** ^1^ Cavendish Laboratory, University of Cambridge, J.J. Thomson Ave., Cambridge CB3 0HE, UK.; ^2^ Department of Chemical Engineering, Pohang University of Science and Technology Pohang, Pohang 790-784, South Korea.; ^3^ State Key Laboratory of Coordination Chemistry, School of Chemistry and Chemical Engineering, Nanjing University, Nanjing 210023, P. R. of China.; ^4^ College of Chemistry and Molecular Engineering, Peking University, Beijing 100871, P. R. of China.; ^5^ Electrical Engineering Division, Department of Engineering, University of Cambridge, 9 JJ Thomson Ave., Cambridge CB3 0FA, UK.; ^6^ Department of Semiconductor Materials Engineering, Faculty of Fundamental Problems of Technology, Wrocław University of Science and Technology, Wybrzeże Wyspiańskiego 27, 50-370 Wrocław, Poland.; ^7^ Institute of Polymer Optoelectronic Materials and Devices, State Key Laboratory of Luminescent Materials and Devices, South China University of Technology, Guangzhou 510640, P. R. of China.; ^8^ Department of Chemistry, Guangdong-Hong Kong-Macao Joint Laboratory of Optoelectronic and Magnetic Functional Materials, Energy Institute and Hong Kong Branch of Chinese National Engineering Research Center for Tissue Restoration & Reconstruction, Hong Kong University of Science and Technology, Clear Water Bay, Kowloon, Hong Kong 999077, P. R. of China.; ^9^ Department of Aerospace Engineering, Khalifa University, Abu Dhabi 127788, UAE.; ^10^ SKKU Advanced Institute of Nanotechnology and Department of Nano Engineering, Sungkyunkwan University, Suwon 16419, Republic of Korea.

## Abstract

Organic thin-film transistors (OTFTs) with ideal behavior are highly desired, because nonideal devices may overestimate the intrinsic property and yield inferior performance in applications. In reality, most polymer OTFTs reported in the literature do not exhibit ideal characteristics. Supported by a structure-property relationship study of several low-disorder conjugated polymers, here, we present an empirical selection rule for polymer candidates for textbook-like OTFTs with high reliability factors (100% for ideal transistors). The successful candidates should have low energetic disorder along their backbones and form thin films with spatially uniform energetic landscapes. We demonstrate that these requirements are satisfied in the semicrystalline polymer PffBT4T-2DT, which exhibits a reliability factor (~100%) that is exceptionally high for polymer devices, rendering it an ideal candidate for OTFT applications. Our findings broaden the selection of polymer semiconductors with textbook-like OTFT characteristics and would shed light upon the molecular design criteria for next-generation polymer semiconductors.

## Teaser

A selection rule for semiconducting polymer candidates for textbook-like thin-film transistors is presented.

## INTRODUCTION

Compared with transistor technologies based on inorganic semiconductors, conjugated polymer-based organic thin-film transistors (OTFTs) (*1*–*3*) have notable advantages including solution processability (*4*) for large-area electronics (*5*, *6*), intrinsic flexibility/stretchability (*7*, *8*) to enable new form factors for electronics, and biocompatibility for various biomedical applications (*9*–*13*). Real-world applications require high field-effect mobilities (*14*); however, another characteristic that is equally important but often overlooked is a robust, ideal transistor behavior that can be well described by standard transistor equations (*15*). Demonstration of these ideal transistors with polymer semiconductors requires a low degree of energetic disorder (*16*–*20*), optimized semiconductor-dielectric interfaces (*21*, *22*), and minimization of contact resistances (*23*). A useful parameter to assess the ideality of a transistor is the reliability factor introduced by Choi *et al.* (*15*), which indicates how well the device can be described by an ideal transistor equation with a gate voltage-independent mobility value. A value of 100% would correspond to a device that is perfectly described by the ideal transistor equations. Unfortunately, most polymer OTFTs exhibit reliability factors <70% because of various reasons including large contact resistance, charge trapping at the semiconductor/dielectric interface, and disorder-induced band tail states, and it is rare to observe the coexistence of relatively high mobility (> 0.1 cm^2^ V^−1^ s^−1^) and ideal charge transport behavior (reliability factors close to 100%) in polymer systems. This creates complexities in circuit design, prevents achieving linear amplification with small signal distortion and constant gain, and causes unpredictable propagation delays (*24*, *25*), thereby hindering applications of OTFTs in areas such as biosensing and integrated digital circuits.

Several families of polymer semiconductors have been reported to exhibit near-ideal transistor behavior: These include the benchmark p-type donor-acceptor copolymer family based on indacenodithiophene-*co*-benzothiadiazole (IDT-BT) and its derivatives, such as IDTT-BT and IDTIDTT-BT shown in fig. S1 and section S6. These polymers exhibit a very low degree of energetic disorder due to their planar and rigid backbone and show nearly gate-independent mobility from room temperature to 200 K (*16*) despite having a low degree of crystallinity (*26*). Another example is the benchmark n-type polymer poly{[*N*,*N*′-bis(2-octyl-dodecyl)-1,4,5,8-naphthalenedicarboximide-2,6-diyl]-alt-5,5′-(2,2′- bithiophene)} (N2200) (*27*). However, unlike IDT-BT, for N2200-based OTFTs a gate voltage–independent mobility has only been observed at room temperature in devices prepared under certain conditions (*28*, *29*). Hence, it would be both fundamentally interesting and practically important to develop an in-depth understanding of the factors that inhibit the demonstration of ideal characteristics and to expand the library of polymer semiconductors exhibiting such ideal transistor behavior.

**Fig. 1.**
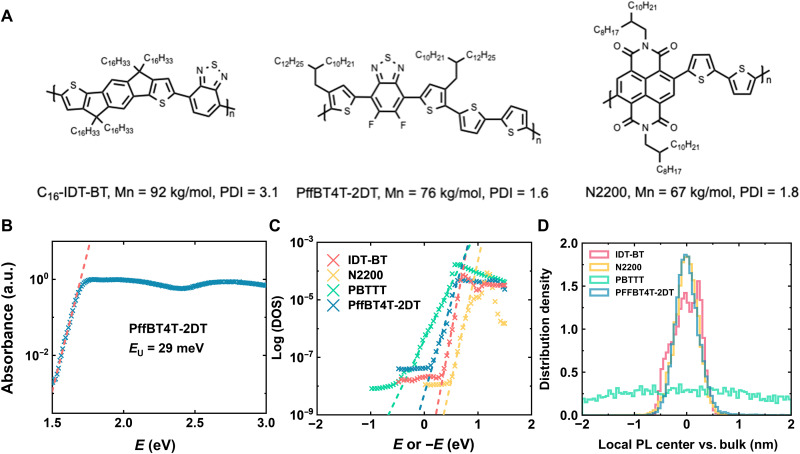
Molecular structures and energetic disorder of conjugated polymers. (**A**) Molecular structures of IDT-BT, PffBT4T-2DT, and N2200. (**B**) PDS spectrum of PffBT4T-2DT thin film with extracted *E*_U_. a.u., arbitrary units. (**C**) ER-EIS of four different polymers (IDT-BT, N2200, PBTTT, and PffBT4T-2DT). For p-type IDT-BT, PBTTT, and PffBT4T-2DT, their highest occupied molecular orbital bands are probed; for n-type N2200, its lowest unoccupied molecular orbital band is probed. The characteristic band tail broadening energy is defined as the inverse of the slope. (**D**) Distribution of local PL COM compared with bulk PL COM of four different polymers.

Here, we report a detailed structure-property relationship study of the semicrystalline donor-acceptor polymer poly[(5,6-difluoro-2,1,3-benzothiadiazole-4,7-diyl)[3,3″‘-bis(2-decyltetradecyl)[2,2′:5′,2″:5″,2″‘-quaterthiophene]-5,5″‘-diyl]] (PffBT4T-2DT) in comparison with IDT-BT and N2200 (Fig. 1A) as well as other polymers (fig. S1). We demonstrate that PffBT4T-2DT exhibits near-ideal transistor behavior with a relatively high, gate voltage–independent hole mobility approaching 0.5 cm^2^ V^−1^ s^−1^, a small threshold voltage and contact resistance, and a near-perfect reliability factor (~100%) in contrast to most reported polymer OTFTs. We observe direct evidence of low excitonic energetic disorder in photothermal deflection spectroscopy (PDS) measurements (*30*, *31*) and of low spatial heterogeneity of emission spectra from nanoscale photoluminescence (PL) mapping. On the basis of our observations, we propose that an effective strategy to realize high-performance polymer OTFTs with ideal behavior would be to optimize materials/devices at different levels: At the single-chain level, a rigid backbone with a small variation of torsional angle is highly desirable to reduce the intrachain energetic disorder, while when sufficient interchain short-contact points (*32*, *33*) or short-range aggregation (*34*) exist, an energetically uniform landscape across a device’s channel can reduce the charge density dependence of mobility.

## RESULTS

### Possible origins of ideal transistor behavior

Previous work has studied some of the factors leading to ideal transistor behavior and that make IDT-BT and N2200 different from other polymers with less ideal OTFT characteristics. In terms of chain conformation, IDT-BT has a near-torsion-free, highly rigidified, and flat backbone that enables substantial polaron delocalization. In contrast, in N2200 a large dihedral angle exists between neighboring naphthalene-di-imide (NDI) and thiophene (T) units, and in *n*-channel OTFTs based on N2200, negative polarons tend to be localized on the NDI cores (*35*). Despite these differences in chain conformation, both polymers exhibit low energetic disorder: On the basis of PDS measurements, IDT-BT has the lowest energetic disorder (24 meV) among all the polymer semiconductors known so far, and N2200 also has a relatively small energetic disorder (around 30 meV) (*36*). Theoretical investigations have suggested that the underlying reason for the low energetic disorder is that the highly rigid monomer units along the backbone exhibit large torsional energy barriers that suppress torsional disorder. While this prediction makes perfect sense for IDT-BT, it may sound counterintuitive for N2200 with nonplanar backbone. In the case of N2200, the rigidity of the backbone is achieved by locking the nonplanar chains in a quasi-frozen conformation (*37*). As a result, although the average torsional angle between the NDI and the thiophene units in N2200 is as large as 38°, the variation of torsional angle (or the torsional disorder) is much smaller than other nonplanar polymers such as PBTTT (*16*). Because the torsion angle modulates the intrachain couplings, a constant nonplanar torsion angle will reduce the coupling strength but does not introduce spatial variations in the couplings, which is beneficial for preserving long-range order and may still lead to high delocalization. The low intrachain energetic disorder at the molecular level is thus believed to be critical for ideal characteristics in OTFTs that are otherwise well optimized.

Here, we are interested in studying charge transport in polymer semiconductors that share the near ideal OTFT characteristics of IDT-BT and N2200. One promising candidate is PffBT4T-2DT, a donor-acceptor polymer that shares the same backbone structure but has slightly longer side chains than the widely studied organic photovoltaic material, poly[(5,6-difluoro-2,1,3-benzothiadiazol-4,7-diyl)-alt-(3,3-di(2-octyldodecyl)-2,2′;5′,2″;5″,2‴-quaterthiophen-5,5-diyl)](PffBT4T-2OD) that exhibits high power conversion efficiency (11%) (*38*). This side-chain arrangement makes solution processing of PffBT4T-2DT OTFTs easier and induces a lower crystallinity than PffBT4T-2OD, while keeping its interesting temperature-dependent aggregation behavior and flat backbone structure due to the fluorinated BT unit (*38*, *39*). PffBT4T-2DT features a flat backbone and a small variation of torsional angle, implying low energetic disorder and effective intrachain charge transport, while the strong aggregation behavior at room temperature could potentially promote interchain charge transport. By optimizing the dip-coating process (*34*), monolayer PffBT4T-2DT transistors with mobility on the same order of amorphous silicon devices was achieved. However, to date, various details regarding the structure-property relationship of thin films in this promising material are still lacking, and a better understanding of the charge transport process in this material is needed.

### Characterization of PffBT4T-2DT thin films

PffBT4T-2DT was synthesized on the basis of the same procedure from previous publications (*38*, *40*), with related properties summarized in table S1 and the thin-film ultraviolet-visible (UV-Vis) spectrum presented in fig. S2. To investigate the energetic disorder of the polymer, we used PDS measurement to probe the broadening of the joint density of states (JDOS) near the band edge (Fig. 1B). A sharp bandgap edge is a hallmark for low energetic disorder in the JDOS and reflects a backbone conformation with low torsional disorder. To better understand the experimental measurements, we performed combined density functional theory and molecular dynamics calculations (section S3) and obtained a relatively narrow distribution of torsion angles for PffBT4T-2DT (fig. S5) of 20.1 ± 10.9° for the angle between the benzothiadiazole and the thiophene units. The width of this distribution is very similar to N2200 (38.2 ± 10.7°) and slightly larger than IDTBT (5.2 ± 4.0°) (*16*). This is fully consistent with PDS measurements of the Urbach energy, *E*_U_, which quantifies the width of the excitonic density of states (DOS) below the optical bandgap and hence the degree of energetic disorder within the system. A low *E*_U_ of 29 meV is extracted for thin PffBT4T-2DT films, slightly higher than the thermal fluctuation at room temperature (*k*_B_*T* = 25.8 meV, *k*_B_ is the Boltzmann constant). To the best of our knowledge, this *E*_U_ value is among the lowest in conjugated polymers, marginally higher than the model polymer IDT-BT (24 meV) (*16*) and very close to N2200 (30 meV) (*29*). Such a low *E*_U_ is beneficial not only to achieve ideal transistor behavior but also to reduce the number of recombination centers that can lead to nonradiative recombination losses in optoelectronic devices. For example, it has been demonstrated that the PffBT4T-2DT–based organic solar cells can achieve an ultrasmall voltage loss of 0.5 V because of its low-energy disorder and the suppression of nonradiative recombination (*41*).

Taking one step further, energy-resolved electrochemical impedance spectroscopy (ER-EIS) (*42*) is used to probe the DOS for charge carriers directly, rather than the JDOS investigated in PDS. Different degrees of DOS broadening can be directly visualized through this technique. Herein, we also compare PBTTT [terrace phase (*43*)], which is a well-known highly crystalline polymer with large energetic disorder, to the three low energetic disorder polymers we investigated (deposition condition of polymer films is presented in the Materials and Methods). We find that PBTTT indeed exhibits broader tail states (the inverse of the logarithmic slope of the band tail is 108 meV) than PffBT4T-2DT (68 meV), IDT-BT (43 meV), and N2200 (58 meV) (Fig. 1C). Note that the DOS obtained from ER-EIS is a combination of the intrinsic properties of polymers and the influence of the electrolyte (e.g., ion intercalation and polymer swelling), and the latter contribution does not exist in PDS, which can explain the qualitative agreement but quantitative discrepancy in energetic disorder.

In addition to low-intrachain energetic disorder at the molecular level, an energetically uniform landscape over the relevant mesoscopic length scales relevant for devices is also essential for achieving ideal behavior in OTFTs. Such a uniform energetic landscape might be easy to achieve for the IDT-BT family of materials, which has a low degree of crystallinity with very broad π-π stacking and large paracrystallinity values *g* ≈ 25% (*16*) and exhibits only nanocrystalline order and local chain alignment on 10-nm length scales (*44*). In the following, we refer to the microstructure of IDT-BT as being near amorphous. In the semicrystalline polymers (N2200, PffBT4T-2DT, and PBTTT) that exhibit much lower paracrystallinity values in their π-π stacking, it is important to investigate this question as the formation of crystalline domains with different orientations of chain alignment separated by grain boundaries with more disordered microstructure can influence the charge transport on length scales of >100 nm. To investigate this, we use state-of-the-art hyperspectral microscopy to directly map the nanoscale PL heterogeneity of targeted thin films and to analyze the statistic distribution of local PL center-of-mass (COM) positions compared with the macroscopic PL from the bulk (fig. S3). It is clear from Fig. 1D that PffBT4T-2DT emits homogeneously across the film, with an impressively narrow variation in local PL COM (SD σ = 0.24 nm) compared with the bulk of the sample. Its local PL COM variation is even slightly narrower than the near-amorphous polymer IDT-BT (σ = 0.25 nm) but slightly wider than N2200 (σ = 0.22 nm). In addition, IDT-BT, PffBT4T-2DT, and N2200 all show substantially narrower distributions compared with PBTTT (σ = 2.5 nm). One possible origin for the wide distribution in PBTTT could be variations in chain alignment direction and/or molecular packing over length scales of several hundred nanometers (*45*). Because PL mapping directly reflects the spatial heterogeneity of polymer bandgap and hence could be correlated with local nonoptimal electronic properties of investigated thin films (*46*), this result confirms more uniform energetic landscapes in films of IDT-BT, N2200, and PffBT4T-2DT compared with PBTTT.

**Fig. 2.**
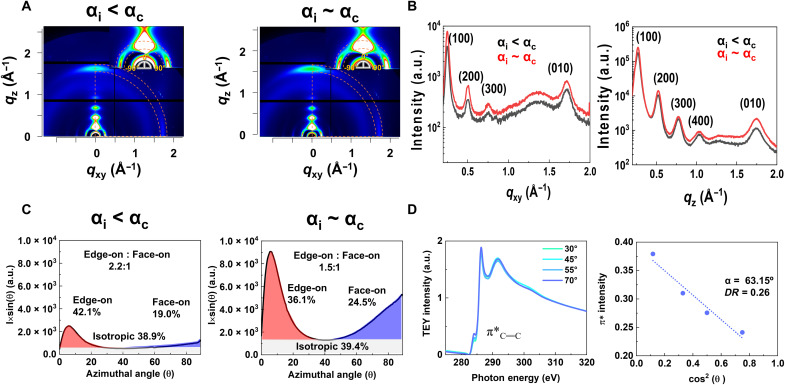
Analysis of GIWAXS and carbon K-edge NEXAFS spectra of PffBT4T-2DT films. (**A**) 2D GIWAXS patterns of spin-coated PffBT4T-2DT films (α_c_ = critical angle of the thin film, α_i_ = incidence angle of the x-ray beam). (**B**) Corresponding GIWAXS line profile along the in-plane/out-of-plane directions. (**C**) Population ratio analysis of edge-on, face-on, and isotropic crystals. (**D**) Total electron yield (TEY) mode NEXAFS spectra with four incidence angles (θ) for the top surface of PffBT4T-2DT films (left) and the π* NEXAFS spectra intensity as a function of cos^2^(θ) with linear fitting (right).

To investigate the possible microstructural origin of low spatial PL heterogeneity, we performed grazing-incidence wide-angle x-ray scattering (GIWAXS) and near-edge x-ray absorption fine structure (NEXAFS) experiments on spin-coated PffBT4T-2DT films. The two-dimensional (2D) GIWAXS patterns in Fig. 2A correspond to two incidence angles, namely, α_i_ ~ 0.08° (α_i_ < α_c_) and α_i_ ~ 0.11° (α_i_ ~ α_c_), probing the microstructure of the topmost surface and deeper into the bulk of the film, respectively. PffBT4T-2DT has a semicrystalline structure, with a preferential edge-on texture. From the in-plane and out-of-plane line profiles in Fig. 2B, several orders of alkyl-stacking (h00) peaks as well as the π-π stacking (010) peak are clearly observable in both directions, with lattice spacing parameters summarized in table S2. From the angle-resolved measurements, we could see a difference in terms of the population ratio between the edge-on and face-on crystallites (Fig. 2C): For the topmost surface, the ratio of edge-on to face-on crystals is 2.2, while for the bulk the ratio is 1.5, revealing a higher degree of edge-on texture at the surface. To further investigate the backbone orientation for the PffBT4T-2DT film, NEXAFS spectra (*47*) were collected with four incident angles (θ between the x-ray and the substrates) (Fig. 2D). The intensity of 1 s-π* transition increases monotonically with increasing θ, and from the linear fit of 1 s-π* transition intensity against cos^2^(θ), the transition intensity at 0° (*I*_0_) and 90° (*I*_90_) were extrapolated, with dichroic ratio $DR=\frac{I_{90}-I_{0}}{I_{90}+I_{0}}$ calculated to quantify the backbone orientation. *DR* = 0.26 is obtained on the basis of the extrapolation results, further confirming a highly edge-on orientation of the PffBT4T-2DT film surface. To quantify the microstructural disorder of PffBT4T-2DT thin films, we calculate the paracrystalline disorder parameter *g* (see Materials and Methods) (*48*). The π-π stacking direction shows typical *g* values for semicrystalline polymers (in-plane, 11.7%; out-of-plane, 12.1%), with the stacking distance of 3.66 and 3.59 Å, respectively. These *g* values indicate that PffBT4T-2DT has higher crystallinity than IDT-BT and is similar to N2200 (the *g* values along the π-π stacking direction are 25% for IDT-BT and 11 to 19% for N2200) (*28*, *49*, *50*), while the more crystalline PBTTT polymer shows a π-π stacking *g* value of merely 7.3% (*48*).

### OTFTs based on PffBT4T-2DT

**Fig. 3.**
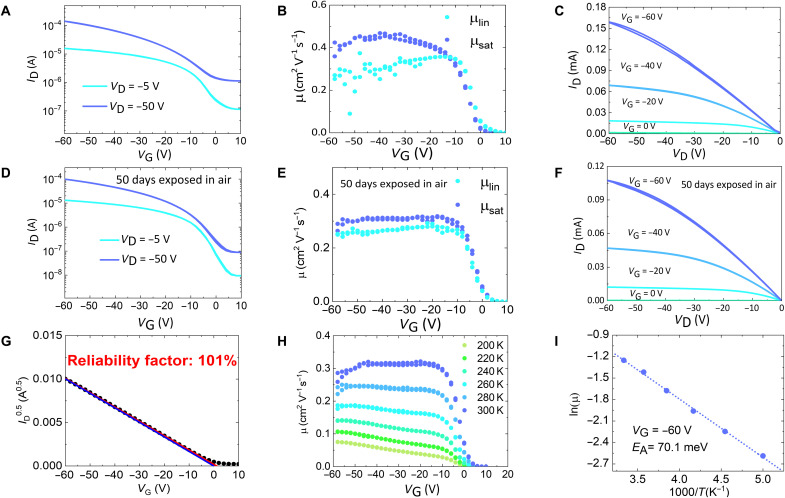
Performance of OTFTs based on PffBT4T-2DT. (**A** and **D**) Linear and saturation transfer curves measured on a representative top-gate, bottom-contact OTFT (*L* = 20 μm, *W* = 1 mm) fabricated from spin-coated, as cast PffBT4T-2DT films. (**B** and **E**) Linear and saturation mobility calculated from the transfer curves measured on the same device. (**C** and **F**) Output curves measured on the same device. (D) to (F) Transfer curves, mobility, and output curves measured after 50 days of air exposure. (**G**) Reliability factor of the device shown in (D): The red line is the linear fit of the transfer curve, and the blue line represents an ideal transistor showing the same maximum current but following the standard transistor equations. (**H**) Temperature-dependent saturation mobility calculated from the transfer curves measured on the same device. (**I**) Activation energy of device extracted from the temperature-dependent saturation mobility at *V*_G_ = −60 V.

The charge transport physics of PffBT4T-2DT was investigated by performing electrical measurements on top-gate, bottom-contact OTFTs (see Materials and Methods for fabrication details). The linear and saturation transfer curves of a representative device are shown in Fig. 3A, with gate voltage–independent saturation mobility approaching 0.5 cm^2^ V^−1^ s^−1^ shown in Fig. 3B. The desirable linear relationship at low drain voltage can be observed in the output characteristics (Fig. 3C), suggesting small contact resistance, which is further confirmed by gated four-probe OTFT measurement (fig. S6). The device also shows impressive air stability (Fig. 3, D to F) and ideal mobility plateau for both linear and saturation mobility (Fig. 3E). The ideal OTFT characteristics indicate a low level of energetic disorder and small amounts of trap states in the PffBT4T-2DT films, similar to the previously reported model polymer IDT-BT and N2200. It is worth noting that this device has a reliability factor close to 100% (Fig. 3G) as a result of the mobility plateau and near-zero threshold voltage, and more importantly, this value is higher than the reliability factors in other polymer OTFTs exhibiting gate voltage–independent mobility reported in the literature (Table 1 and table S3).

**Table 1.** Figure of merit summary of four polymers investigated.

Polymer	*E*_U_ (meV)	ER-EIS disorder (meV)	PL mapping COM width (nm)	*g* (%)	μ (cm^2^ V^−1^ s^−1^)	Reliability factor (%)
IDT-BT	24 (*16*)	43	0.25	25	~1	79 (*16*)
N2200	30 (*36*)	58	0.22	11–19	~0.2	88 (*28*)
PffBT4T-2DT	29	68	0.24	12	~0.5	101
PBTTT	47 (*16*)	108	2.5	7	<0.1	64 (*16*)

We further performed a temperature-dependent charge transport study on the same device. The saturation transfer curves between 200 and 300 K are presented in fig. S7 and fitted to *I*_D_ ∝ (*V*_G_ − *V_th_*)^γ^, with extracted saturation mobility presented in Fig. 3H. For an ideal transistor, the exponent γ should be 2, while for a 2D disordered system with an exponential DOS, γ is temperature dependent and follows the form of $\gamma =\frac{T_{0}}{T}+1$, where *T*_0_ is the characteristic width of the DOS (*T*_0_ > *T*) (*36*). The PffBT4T-2DT transistor lies in the crossover regime from an ideal transistor to a 2D disordered system with decreasing temperature. At 300 K, the mobility is gate independent, and the transfer curves follow ideal behavior (γ = 2). At lower temperatures, the device starts to deviate from a textbook-like transistor and behaves more like a 2D disordered system (fig. S8). It is worth noting that the extracted *T*_0_ value is ~282 K (~24 meV), lower than most polymer semiconductors except IDT-BT (*36*). This energy scale is even lower than *k*_B_*T* at room temperature, which suggests that the prerequisite for the disorder model is no longer valid at 300 K and might explain why the device behaves ideally at room temperature. We observed very similar temperature-dependent behavior in N2200, which shows a low *T*_0_ value of 270 K (23 meV) and γ ~ 2 at 300 K (fig. S8), indicating similar degrees of energetic disorder in these two systems.

From Fig. 3H, clear thermal activation behavior is observed, with an extracted activation energy around 70 meV presented in Fig. 3I. This level of activation energy is similar to the values of several polymers: IDT-BT OTFTs (61 meV) with the same top-gate bottom-contact architecture and CYTOP dielectric layer (*51*), optimized regioregular poly-3-hexylthiophene (P3HT) (54 to 69 meV) (*52*), and PBTTT (55 to 58 meV) (*53*) devices with bottom gate architecture. Although IDT-BT and PffBT4T-2DT show much lower *E*_U_ compared with regioregular P3HT (50 meV) (*36*) and PBTTT (47 meV) and also notably different morphologies *(**43*, *51*, *54*), their comparable activation energy for OTFTs might indicate that charge transport processes within these polymers are limited by similar factors such as reorganization energy, which has been proposed to play a critical role in determining the activation energy for OTFTs based on N2200 (*35*).

**Fig. 4.**
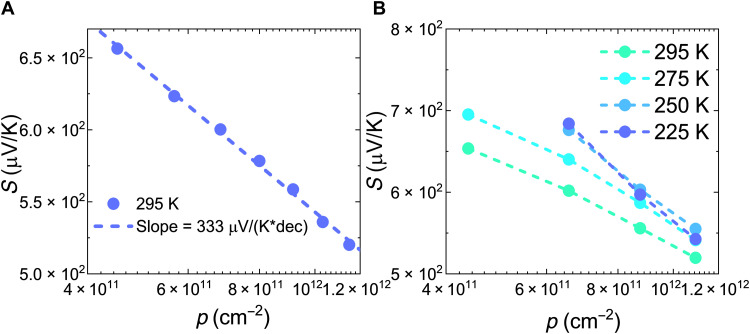
Seebeck measurements of OTFTs based on PffBT4T-2DT. (**A**) Seebeck coefficients versus the logarithm of carrier concentration for PffBT4T-2DT OTFT at 295 K. (**B**) Temperature-dependent Seebeck coefficient versus the logarithm of carrier concentration for PffBT4T-2DT OTFT.

We also performed field-effect–modulated Seebeck coefficient (*S*) measurements on PffBT4T-2DT films as a function of gate voltage (carrier concentration) and temperature using a microfabricated device (*55*), which provides important information on the material’s energetic disorder and the trap DOS (*16*) (see Materials and Methods and section S7 for device fabrication process and working principle). For the range of carrier concentration investigated, the Seebeck coefficient is between 500 and 700 μV/K, which is much higher than *k*_B_/*e* ≈ 86 μV K^−1^ (*e* is the elementary charge). The *S*-log(*p*) plot (Fig. 4A) of the PffBT4T-2DT transistor has a slope of 333 μV K^−1^ decade^−1^. On the basis of a narrow-band model (*16*), this slope can be expressed as (*k*_B_/*e*)ln(10)/(1 − *f*), where *f* is the fraction of trapped carriers (see section S7). We then obtain *f* = 0.4 for PffBT4T-2DT, indicating that at 295 K, most of the charge carriers injected into the OTFT channel would stay in the mobile states and thus indicates the intrinsic low disorder of this polymer. This fraction of trapped charge is larger than IDT-BT (0.3) and N2200 (0.1) but smaller than other semicrystalline polymers such as PBTTT (0.5) and PSeDPPBT (0.7) (*16*). Furthermore, the temperature dependence of the Seebeck coefficient (Fig. 4B) is weak (close to the measurement error of 50 to 100 uV/K) especially at high carrier concentration, which is very similar to the case of IDT-BT, suggesting that the energetic dispersion of thermally accessible states is small and is well within the range of *k*_B_T.

On the basis of the observation of gate-independent carrier mobility and ideal transistor behavior in 3 different families of polymer semiconductors (IDT-BT, N2200, and PffBT4T-2DT), we summarize two empirical rules for the realization of ideal polymer OTFTs. (i) A highly rigid polymer backbone with small variation of torsional angle is preferred because it helps reduce the intrachain energetic disorder. If *E*_U_ is much larger than room temperature thermal energy *k*_B_*T*, charge transport would most likely be limited by the broadening of DOS and hence the field-effect mobility becomes gate dependent. For IDT-BT, *E*_U_ is as low as 24 meV and its ideal transistor behavior survives down to 200 K; for PffBT4T-2DT and N2200, *E*_U_ is slightly higher (~30 meV) but still low enough for ideal transistor behavior to be observed at room temperature. (ii) A spatially uniform microstructure is preferred because gate-independent mobility also requires a smooth energetic landscape for charge transport over mesoscopic, device-relevant length scales. Intuitively, one would expect smooth energetic landscapes in single-crystalline or fully amorphous polymer films, but in reality, it might not be possible to make every polymer as nearly amorphous as IDT-BT (*g* ~ 25%). We show here that a uniform energetic landscape is also achievable for semicrystalline polymers with smaller *g* than IDT-BT along the π-π stacking direction including PffBT4T-2DT (12%) and N2200 (11 to 19%), which might, at least partly, originate from their favorable chain design that makes their electronic structures more resilient to structural disorder and contribute to low energetic disorder both along a polymer chain and across a transistor channel. When these two criteria are met, a textbook-like, gate-independent mobility may be achieved, which is highly preferred over a mobility with stronger gate dependence (even if the maximum mobility values in the latter case may be higher). An ideal gate voltage–independent mobility makes device modeling and circuit design much easier especially for complicated circuitry and is highly desirable for applications requiring linear amplification with minimum signal distortion and constant gain (e.g., biosensing), and for applications requiring reliable and predictable signal propagation delay (e.g., integrated circuits). For example, the oscillation frequency of a ring oscillator decreases with increasing nonlinearity of OTFT transfer curves, and even a small nonlinearity (reliability factor ~80%) can lead to a substantial reduction of oscillation frequency (see fig. S14).

## DISCUSSION

In this work, we have investigated two key factors for achieving ideal behavior in OTFTs, namely, a polymer chain conformation with low intrachain, torsional disorder, and an energetically uniform landscape over device-relevant length scales (more likely achieved in polymers with electronic structures resilient to structural disorder). We show that in addition to near-amorphous polymers like IDT-BT and semicrystalline N2200, these requirements are also fulfilled in semicrystalline PffBT4T-2DT. For PffBT4T-2DT, we obtained thin films with uniform energetic landscapes and observed OTFTs with room-temperature textbook-like performance and reliability factor close to 100%, outperforming many existing polymer OTFTs with higher, maximum mobility values but nonideal characteristics. Our results expand the class of polymers in which ideal OTFT behavior can be observed and provide insights into the molecular design criteria for achieving such textbook-like characteristics in polymer OTFTs, which are beneficial for a broad range of OTFT applications.

## MATERIALS AND METHODS

### Deposition condition of polymer films

#### PffBT4T-2DT

Films were spin-coated at 1200 rpm for 60 s from 60°C anhydrous 1,2-dichlorobenzene solution (5 mg/ml) and then dried at 90°C for 2 min to remove residual solvent.

#### PBTTT

Films [terrace phase (*43*)] were spin-coated at 1500 rpm for 60 s from 90°C anhydrous 1,2-dichlorobenzene solution (10 mg/ml) and subsequently annealed at 180°C for 20 min and then slowly cooled to room temperature.

#### IDTBT

Films were spin-coated at 1500 rpm for 60 s from 60°C anhydrous 1,2-dichlorobenzene solution (10 mg/ml) and then dried at 90°C for 2 min to remove residual solvent.

#### N2200

Films were spin-coated at 1500 rpm for 60 s from 90°C anhydrous 1,2-dichlorobenzene solution (10 mg/ml) and then dried at 90°C for 2 min to remove residual solvent.

All the sample preparation steps were carried out in a nitrogen glovebox using glass pipettes and substrates preheated to the same temperature as the solutions.

### UV-Vis and PDS measurements

To spin-coat the PffBT4T-2DT thin film, PffBT4T-2DT solution was made by dissolving the PffBT4T-2DT into anhydrous 1,2-dichlorobenzene with a concentration of 5 mg/ml and spin-coated on top of the substrates at 1200 rpm/min for 60 s, giving a PffBT4T-2DT layer of around 30 nm. After this step, the sample underwent a 2 min annealing step at 90°C to get rid of the bulk of the solvent. UV-Vis absorption measurement was performed with a SHIMADZU UV-3600i Plus UV-VIS-NIR spectrophotometer. The PDS measurement was performed with a homemade PDS setup based in the Cavendish Laboratory following the same procedure as in (*16*). The Urbach energy, *E*_U_, is extracted by exponentially fitting the subenergy gap tail based on the equation below

$$\alpha (\hbar \omega )∼\exp\left(\frac{\hbar \omega -E_{g}}{E_{U}}\right)$$

### ER-EIS measurements

Polymer films used for ER-EIS were spin-coated on Au-coated glass slides. Ionic liquid 1-Butyl-1-methylpyrrolidinium bis(trifluoromethylsulfonyl)imide ([BMP][TFSI]) was used as the electrolyte. ER-EIS measurements were performed in an N_2_-filled glovebox with a PalmSens4 potentiostat, a Pt counter electrode, and a Ag quasi-reference electrode. The AC voltage signal had a frequency of 0.5 Hz and an rms value of 50 mV, and the sweep rate of the DC voltage was 2 mV/s.

### Hyperspectral PL measurements

The PL emission maps were acquired using a wide-field hyperspectral microscope (IMA VISTM, Photon Etc.) with a calibrated low-noise silicon charge-coupled device (CCD) camera. A 405-nm continuous wave laser was focused on the sample surface with an excitation density of ~10 W cm^−2^, and the reflected laser beam was filtered off by 420-nm long-pass filter. Samples were encapsulated before measurement. Details of the procedure could be seen in (*56*). The initial PL maps had 1024 × 1024 resolution, and the area of a pixel was 66 × 66 nm. Each pixel contained PL spectrum from 600 to 1000 nm with a step size of 2 nm. To improve signals for curve fitting, the boundary of image was cropped out and a 10 × 10 binning was applied on the raw data, resulting a reduction of image resolution from 1024 × 1024 to 100 x 100 (thus a total of 10,000 pixels). COM was calculated using Python SciPy library for local PL curve of each pixel in the PL maps.

### GIWAXS and NEXAFS measurements

The GIWAXS measurement was carried out with Pohang Accelerator Laboratory beamline using an In-Vacuum Undulator 20B source (11.08 keV) and a 2D CCD detector (Rayonix SX165, USA). The incidence angles were 0.08° (α_i_ < α_c_) and 0.11°(α_i_ ~ α_c_). The samples for GIWAXS measurements were fabricated on silicon substrates using the same recipe for the devices. NEXAFS experiments were detected by Scienta R3000 with 10^11^ (photon/s) intensity from a bending magnet-type light source (beam size ~0.7 mm by 1.2 mm) and fully calibrated with Au 4f peak. Angle-resolved NEXAFS spectroscopy was conducted in total electron yield mode to collect the molecular orientation information from the uppermost (~10 nm) surface part of the PffBT4T-2DT thin films.

### Calculation of paracrystalline disorder parameter *g*

To quantify the microstructural disorder of PffBT4T-2DT thin films, we calculate the paracrystalline disorder parameter *g* for the lamellar and π-π stacking along these two directions, which is defined as the statistical SD from its average lattice position according to the following equation

$$g=\sqrt{\frac{{\text{Δ}}_{q}}{2\pi q_{0}}}$$

where ∆*_q_* is the FWHM of a diffraction peak and *q*_0_ is the center position of the same peak.

### OTFT fabrication

Top-gate bottom-contact OTFT architecture is used to fabricate PffBT4T-2DT devices. Photolithographically defined interdigitated source/drain electrodes (3-nm chromium adhesion layer and 17-nm gold layer on top, with channel length *L* = 20 μm and channel width *W* = 1 mm) were created on top of glass substrates (low-alkali 1737F Corning glass), following standard photolithography and lift-off process (*57*). PffBT4T-2DT was spin-coated as described previously (in the “Deposition condition of polymer films” section). CYTOP (500 nm, M-type from AGC Chemicals) was spin-coated on top of the PffBT4T-2DT layer. Last, the 20-nm aluminum gate layer was evaporated on top of the device channel through a shadow mask at low pressure (2 × 10^−6^ mbar) to complete the device fabrication.

### Seebeck device fabrication

The Seebeck device is also based on a top-gated bottom-contact OTFT architecture. After fabricating the source/drain electrodes and spin-coating the PffBT4T-2DT layer, the channel was patterned according to previous publications (*16*). CYTOP (500 nm) was spin-coated on top of the patterned semiconductor, and 20-nm Al was evaporated through a shadow mask to serve as the gate.

### OTFT and Seebeck device testing

An Agilent 4155B Semiconductor Parameter Analyser was used to measure transfer and output curves for the OTFTs. Room-temperature OTFTs were measured in a nitrogen environment (oxygen and moisture level below 3 ppm). For air stability experiments, the device was stored in the air for 50 days. Temperature-dependent OTFT measurements were performed in a Desert Cryogenics probe station. Seebeck measurements (including thermal voltage and temperature sensor calibration measurements) were performed in a Lake Shore CRX-4 K cryogenic probe station with Keithley 2612B and 6430 source-measure units. More details are presented in section S7.

## Supplementary Materials

### This PDF file includes:

Supplementary Text Figs. S1 to S14 Tables S1 to S3 References
