# Supplementary material for: Achieving ideal transistor characteristics in conjugated polymer semiconductors
Source: Sci Adv. 2023 Jun 2;9(22):eadg8659. doi: 10.1126/sciadv.adg8659 (PMC10413658; doi:10.1126/sciadv.adg8659)
Supplement: Supplementary file 1 — Supplementary Text Figs. S1 to S14 Tables S1 to S3 References [file sciadv.adg8659_sm.pdf]

Science Advances
AAAS

Supplementary Materials for

Achieving ideal transistor characteristics in conjugated polymer semiconductors

Mingfei Xiao *et al.*

Corresponding author: Xinglong Ren, xr216@cam.ac.uk; He Yan, hyan@ust.hk; Shangshang Chen, schen@nju.edu.cn; Henning Sirringhaus, hs220@cam.ac.uk

Sci. Adv. **9**, eadg8659 (2023)
DOI: 10.1126/sciadv.adg8659

This PDF file includes:

Supplementary Text
Figs. S1 to S14
Tables S1 to S3
References

Supplementary Materials

Section 1. Additional information of polymers investigated

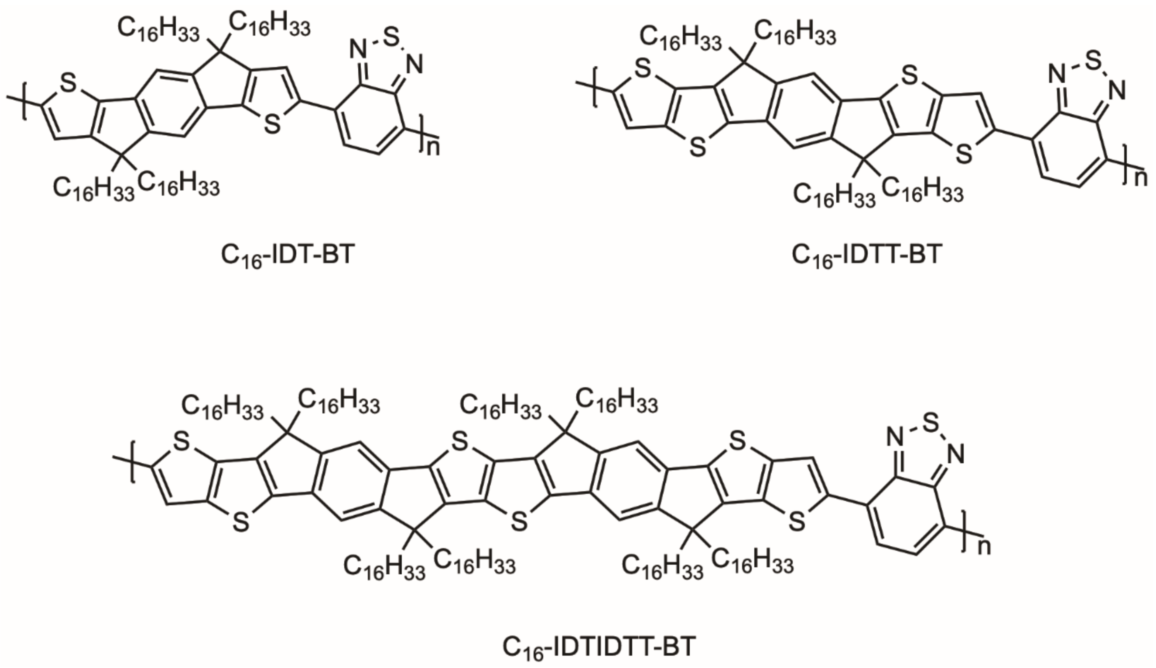

Fig. S1. Molecular structures of polymers belong to the IDT-BT family.

Table S1 Polymer molecular weight, ionisation energies, electron affinities, optical properties

Polymer	M_n (kg/mol)	M_w (kg/mol)	IP^a (eV)	EA^b (eV)	λ^c (nm)	$E_{opt}{}^d$ (eV)
PffBT4T-2DT	75.6	123	-5.18	-3.49	752	1.65

[ab] IP and EA are measured by cyclic voltammetry(*58*)

[c] Thin films were spin-coated on glass substrates, λ is the peak of the first low energy absorption band of the polymer

[d] Estimated optical gap was calculated using onset of the thin-film absorption spectra ($E_{opt} = 1240/\lambda_{onset}$)

Section 2. Optical characterization of PffBT4T-2DT films

UV-Vis measurement

In **Fig. S2,** the absorption spectrum of PffBT4T-2DT thin film is presented. A strong, structured vibronic progression of the internal charge-transfer (ICT) state near the energy-gap edge at at 700 nm, and the π-π* transition at 440 nm each agree well with previous experimental results for the same polymer(*39*). The strong transition electric dipole moment for the 0-0 vibronic peak suggests J-

aggregate behaviour(*59*) which is well-reported for other polymers of this class with planar chains(*60*), low energetic disorder and weak intermolecular coupling(*39*)(*59*).

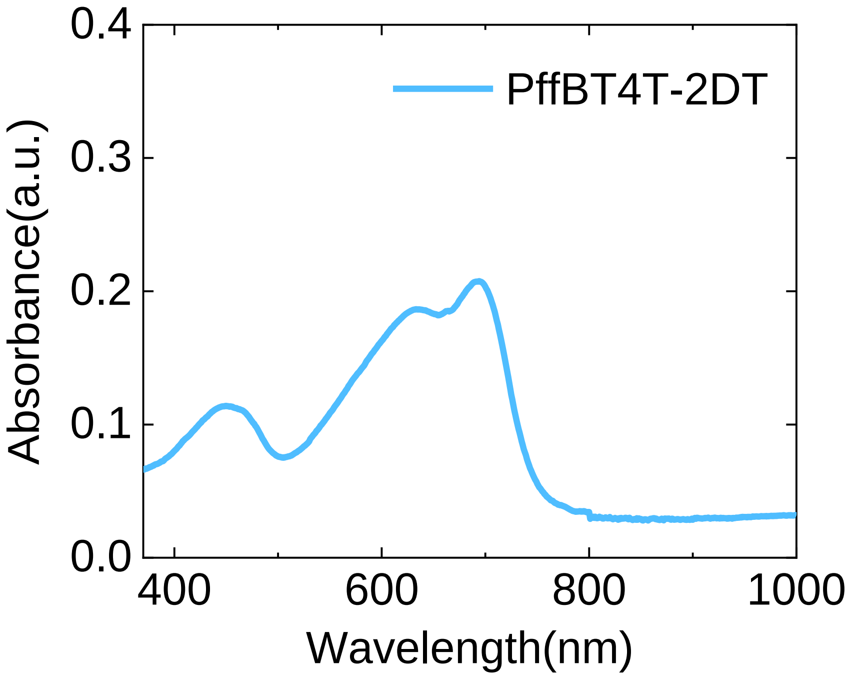

Fig. S2. UV-Vis spectrum of PffBT4T-2DT thin film.

Hyperspectral PL measurements

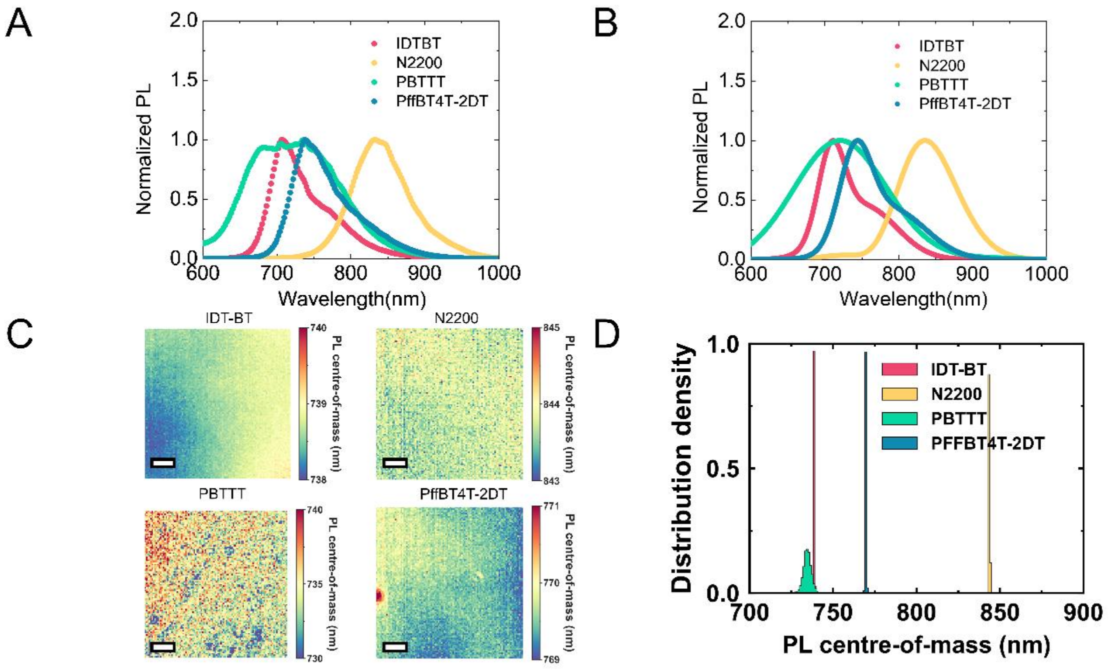

Fig. S3. Study of nanoscale PL heterogeneity of IDTBT, N2200, PBTTT, and PffBT4T-2DT films.

(**A**) Normalized PL spectrum of bulk films. (**B**) Normalized PL spectrum of the first pixel within each sample (pixel on the topleft corner). (**C**) Local PL centre-of-mass maps of polymers. Pixels that cannot be Gaussian fitted are in white and excluded from the statistical analysis, scalebars are 10 µm. (**D**) Distribution of local PL centre-of-mass (10,000 pixels in total, size of each: 660 x 660 nm^2) for all the four materials within the region of interest.

Section 3. Torsional disorder calculations

The torsion potentials between various comonomer units of cationic and neutral PffBT4T-2DT was computed at DFT/B3LYP/def2-TZVP level of theory. All the long alkyl chains were replaced by methyl groups to reduce the computational cost.

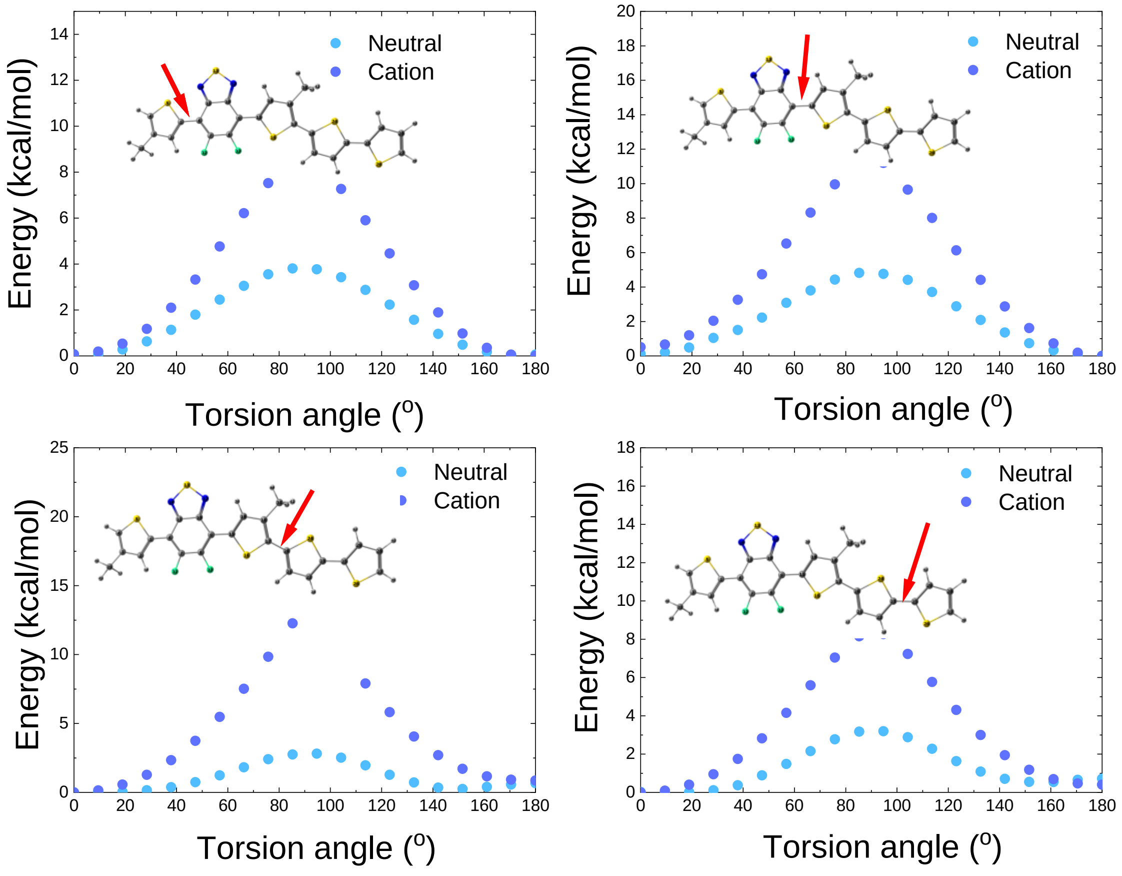

Fig. S4. Calculated torsion potentials of PffBT4T-2DT.

According to our previous procedure(*61*)(*62*), polymer crystal structure of PffBT4T was constructed with optimized monomer structure, interdigitated alkyl side chains, and lattice parameters from GIWAXS experiments. The crystal structure was then fully optimized with periodic-boundary-condition (PBC). A 3×6×8 supercell was built with 3 lamellar layers of 6 π-stacked chains with 8 repeating units. The supercell was subjected to a 50-ps molecular dynamics simulation under NPT ensemble (P = 1×10^5 Pa, T = 500 K) to induce polymer packing disorders, following a 1-ns molecular dynamics at room

temperature under NPT ensemble (P = 1×10^5 Pa, T = 298 K). The equilibrium state was then extracted and visualized. Molecular dynamics calculations were performed with Materials Studio package using the Dreiding force field(*63*), Gasteiger charges(*64*), velocity scale thermostat, Berendsen pressure coupling, and time step of 2 fs.

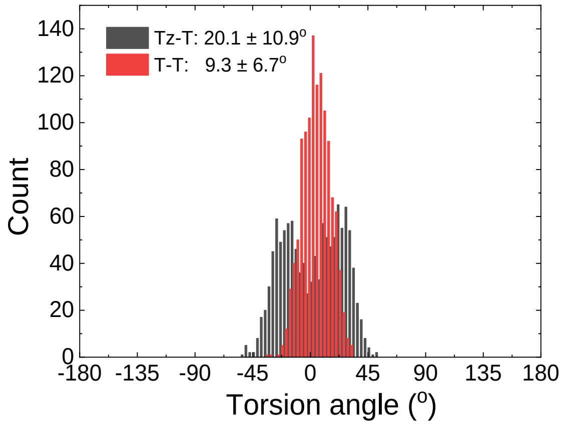

Fig. S5. Distribution of torsion angles along the PffBT4T-2DT backbone.

Tz-T stands for the angle between the benzothiadiazole unit and the thiophene unit which shows a wider distribution, and T-T stands for the angle between tow thiophene units.

Section 4. Crystallographic parameters of PffBT4T-2DT

Table S2 GIWAXS spacing parameters for spin-coated PffBT4T-2DT sample

PffBT4T-2DT						
Crystallographic parameters	In-plane			Out-of-plane		
Lamella packing	(100)	*q* (Å^{-1})	0.25212	(100)	*q* (Å^{-1})	0.27141
		d-spacing (Å)	24.9		d-spacing (Å)	23.2
		FWHM (Å^{-1})	0.03694		FWHM (Å^{-1})	0.04788
		g (%)	15.3		g (%)	16.8
π–π stacking	(010)	*q* (Å^{-1})	1.7156	(010)	*q* (Å^{-1})	1.74884
		π–π stacking (Å)	3.66		π–π stacking (Å)	3.59
		FWHM (Å^{-1})	0.14651		FWHM (Å^{-1})	0.16136
		g (%)	11.7		g (%)	12.1

Section 5. More details of OTFTs based on PffBT4T-2DT

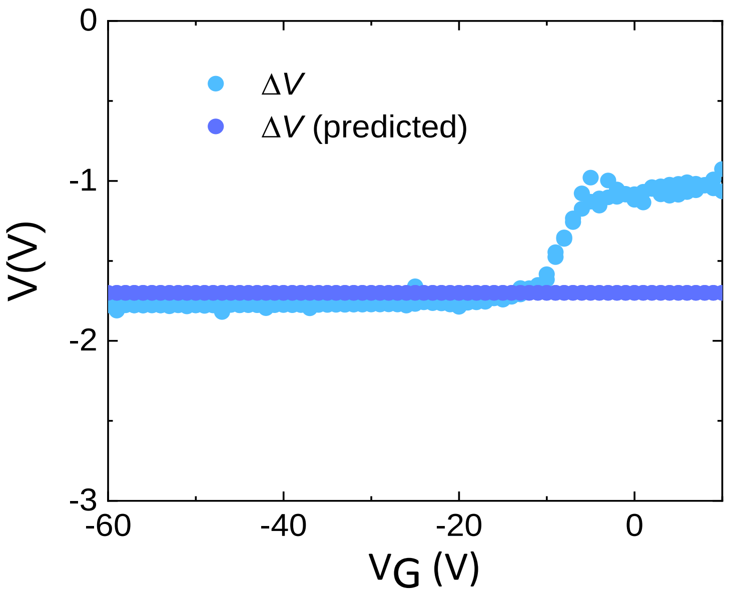

Fig. S6. Comparison between the theoretical and the measured voltage difference between the two voltage probes (Δ*V*) in a gated four probe transistor measurement.

For the gated four probe transistor measurement (**Fig. S6**), the applied drain voltage is –5 V, and the channel length *L* and the distance between 2 voltage sensing probes Δ*L* are 420 μm and 140 μm respectively. Assuming that the potential drops linearly between the source and the drain, the theoretically predicted Δ*V* should be –1.67 V. The measured value, however, is –1.77 V, the magnitude of which is even slightly larger than the theoretical value. If the measured value is used to calculate the contact resistance, we would get a small but negative contact resistance for this device, which is obviously not reasonable. One possible explanation of the negative apparent contact resistance is that the gradual channel approximation is not 100% valid (i.e., the potential drop across the channel is slightly non-linear). The observation of the negative apparent contact resistance suggests that the true contact resistance should be negligible compared with the channel resistance, because the existence of any noticeable contact resistance would lead to a smaller magnitude of Δ*V* than the theoretically predicted value.

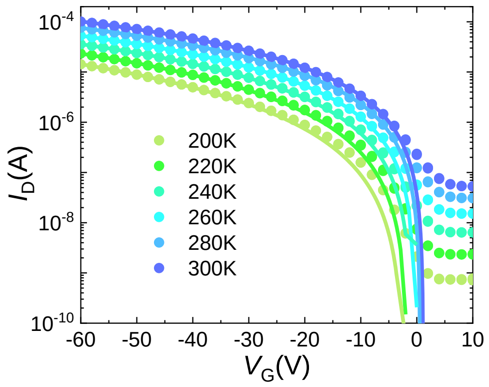

Fig. S7. Fitting of low T saturation transfer curves.

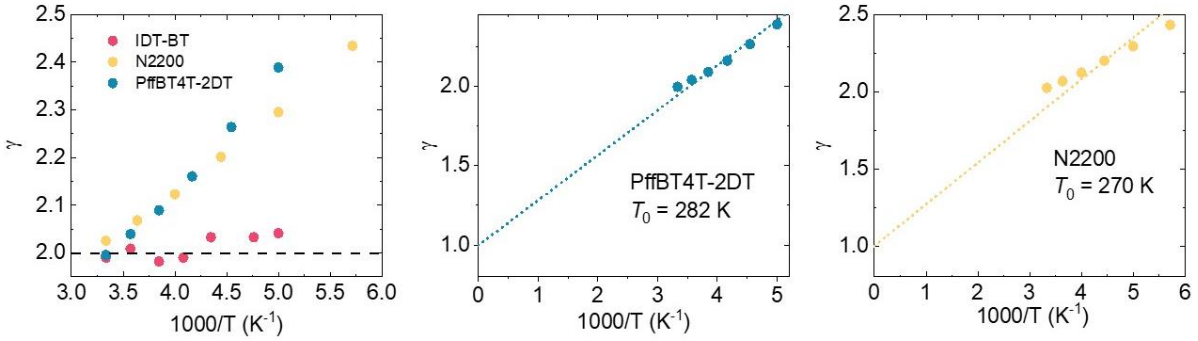

Fig. S8. Extracted values of *γ* and T_0 from T-dependent transfer characteristics.

Data for IDT-BT and N2200 are reproduced from Ref (*16*) and (*28*) respectively.

Section 6. OTFTs based on other polymers

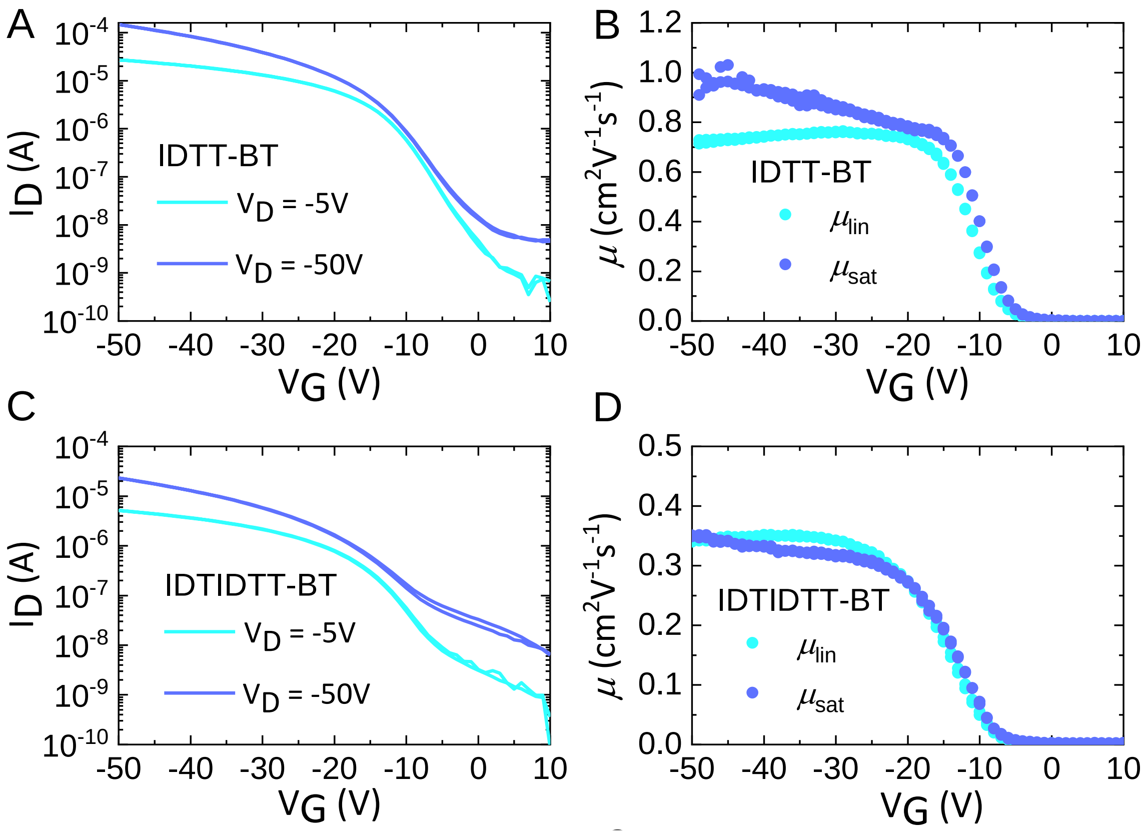

Fig. S9. Performance of OTFTs based on IDTT-BT and IDTIDTT-BT.

Linear and saturation transfer curves measured on a representative top-gate, bottom-contact OTFT (*L*= 20 μm, *W*=1 mm) fabricated from the spin-coated, as cast (**A**) IDTT-BT film, and (**C**) IDTIDTT-BT film. Linear and saturation mobility calculated from the transfer curves measured on the same device for (**B**) IDTT-BT, and (**D**) IDTIDTT-BT.

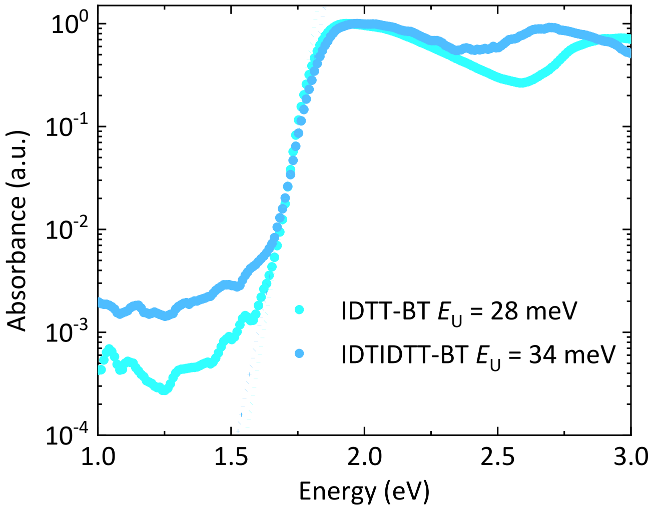

Fig. S10. PDS spectrum of IDTT-BT and IDTIDTT-BT thin film with extracted E_U.

Table S3 Reliability factors for different polymer OTFTs with V_G-independent mobility. Note that the difference in reliability factor is mainly due to the difference in threshold voltage.

Polymer	Reliability factor	Reference
IDT-BT	79%	(*16*)
N2200	88%	(*28*)
IDTT-BT	73%	This work
IDTIDTT-BT	72%	This work
PffBT4T-2DT	101%	This work

Section 7. More details of Seebeck measurements

The schematic and optical image of the measured Seebeck device is shown in **Fig. S11.** A microfabricated heater and two temperature sensors (sensors at the hot and the cold end of the device, respectively) are positioned along the OTFT channel, while the two sensors also work as the source and drain electrodes of the OTFT device. The linear and saturation mobility of the measured device at room temperature is shown in **Fig. S12**. The mobility measured from the Seebeck device shows a lower value compared with the one measured from normal OTFTs. This is likely due to the photolithographic patterning process, which was used to pattern the polymer film to realize this architecture (*65*) and which negatively affects the performance of the PffBT4T-2DT layer. The Seebeck coefficient S is determined by measuring the electromotive force EMF (ΔV) across the material when a temperature differential ΔT is generated by the heater along the same direction, and is given by the equation below:

$$S = \frac{\Delta V}{\Delta T}$$

In this experiment, the temperature gradient is created by resistive heating (applying a voltage to the on-chip heater). The temperature difference between the hot side and the cold side is calculated by first measuring the temperature coefficient of resistance ($\frac{dR}{dT}$, **Fig. S13A**) of the metal temperature sensors, and then obtaining the heating power (P) dependence of sensor resistance ($\frac{dR}{dP}$, **Fig. S13B**). The thermal voltage is also measured under the same heating power ($\frac{dV}{dP}$, **Fig. S13C**). Consequently, the final expression of S is:

$$S = \frac{\frac{dV}{dP}}{\frac{(\frac{dR}{dP})hot}{(\frac{dR}{dT})hot} - \frac{(\frac{dR}{dP})cold}{(\frac{dR}{dT})cold}}$$

A narrow-band model is used to interpret the Seebeck coefficient based on the following equation(*66*):

$$S = \frac{k_\mathrm{B}}{e}\ln\left(\frac{N - n_c}{n_c}\right) + \frac{k_\mathrm{B}}{e}\ln(2) + \alpha_\mathrm{vib}$$

The first contribution is related to the change of the entropy of mixing associated with adding a carrier into the density of thermally accessible transport states, with N denoting the total number of available states and n_c the mobile carrier concentration. The second term is the entropy change due to spin degeneracy, while the third term is the entropy change arising from the molecular vibration(*67*). Within the equation, only the first term is carrier concentration-dependent, and since in the case of an OTFT $n_c \ll N$, the plot of S versus the logarithm of n_c would give a straight line with the slope of $(k_B/e)\ln(10)$ = 198 µV K^{-1} $decade^{-1}$. In reality, most polymer systems exhibit slope larger than 198 µV K^{-1} $decade^{-1}$. This discrepancy could be reconciled by taking into account the proportion f of trap states within all the thermally accessible sites, which gives $n_c = n(1-f)$. Then the slope of the S-log(n) plot is modified to be $-(k_B/e)\ln(10)/(1-f)$.

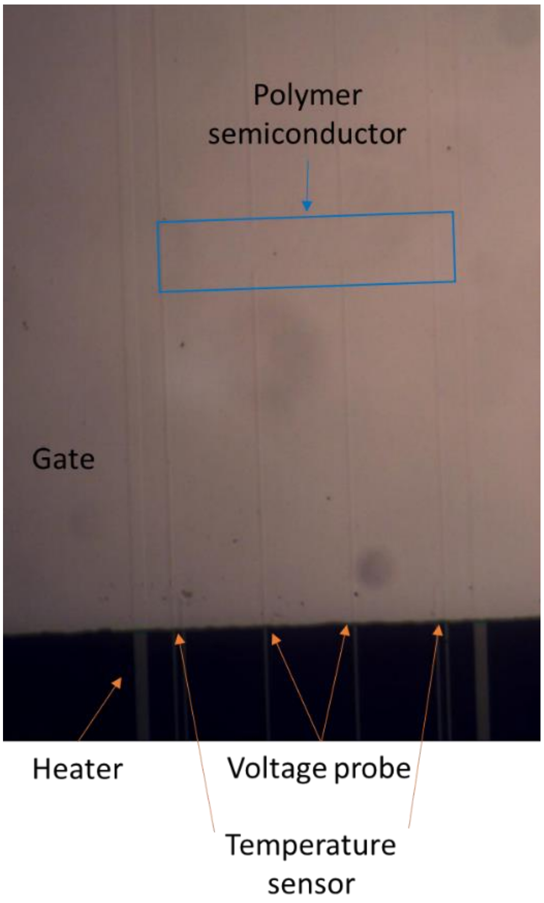

Fig. S11. Optical image of the gated-Seebeck device.

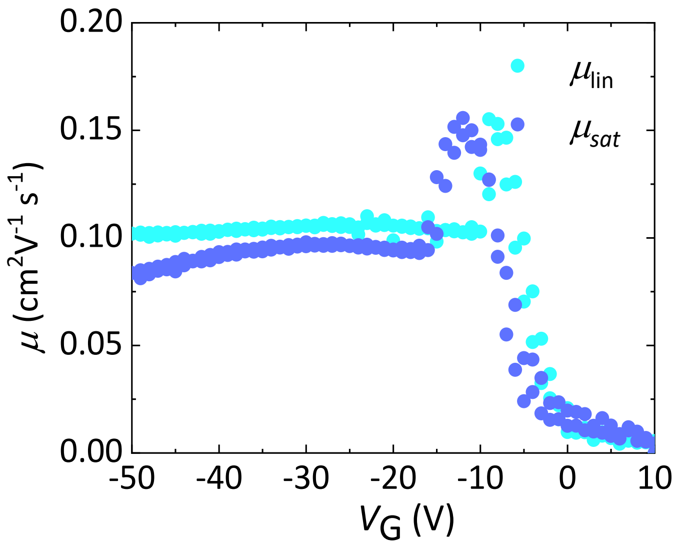

Fig. S12. Room-temperature linear and saturation mobility of the top-gate, bottom-contact OTFT (*L*= 420 μm, *W*=105 mm) fabricated with the patterned PffBT4T-2DT layer used for the Seebeck measurements.

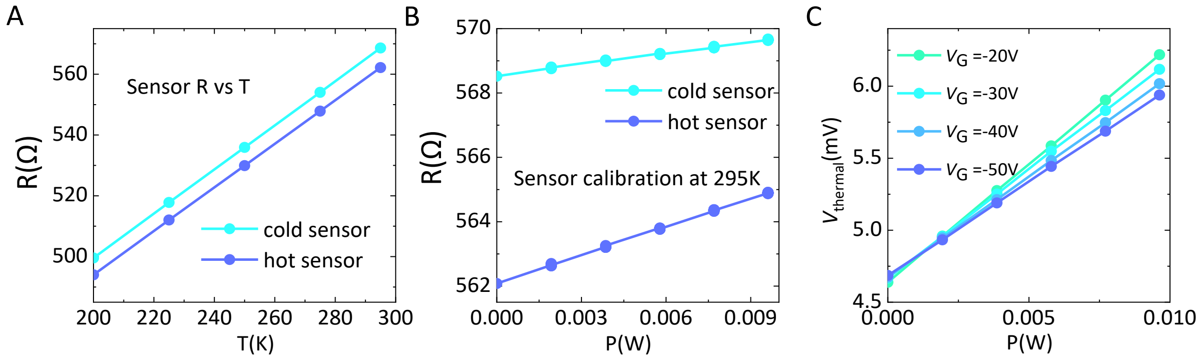

Fig. S13. Characterization of cold and hot end sensors.

(A) Temperature-resistance relationship of cold and hot end sensor. (**B**) Heater power-resistance relationship of cold and hot end sensor. (**C**) Heater power-thermal voltage relationship at different gate voltage.

Section 8. Ring oscillator simulation

We evaluated the critical impacts of reliability factor on circuit performance by simulating the oscillation frequency of seven-stage ring oscillators using Technology Computer-Aided Design (TCAD)(*68*)(*69*). The schematic of seven-stage ring oscillator in simulation is shown in **Fig. S14A**, constructed by cascading seven inverters with p-type OTFTs acting as both the driver and the load. Six seven-stage ring oscillators were simulated based on six types of p-type OTFTs with identical drain current at -50 V but increasing non-linearity (reliability factors ranging from 101% to 20% (**Fig. S14B**)). OTFTs with reliability factor of 100% are considered as ideal transistors with gate independent mobility, while 101% ones resembling the OTFTs based on PffBT4T-2DT of this work and 80% being the maximum value of most polymer-based OTFTs exhibiting certain levels of non-linearity reported in literature. The driver and load OTFTs have the same channel length but different channel width, denoted as W_{driver} and W_{load} respectively. However, the ratio between W_{driver} and W_{load} has minimum effects on the relative frequency of oscillators based on OTFTs with different reliability factors compared with that based on ideal OTFTs (**Fig. S14C**). The output waveforms of oscillators based on OTFTs with different reliability factors are shown in **Fig. S14D** at supply voltage of 50V, with the frequency of the oscillator of 101% reliability factor reaching 49.434 kHz and that of 20% reliability factor close to 0. Voltage dependent frequencies of oscillators are shown in **Fig. S14E**, showing clear positive correlation between oscillation frequency and supply voltage as well as positive correlation between oscillation frequency and reliability factor. To compare the performance of ring oscillators with different reliability factors, their oscillation frequencies are normalized by the frequency of the oscillator with 100% reliability factor ($f/f_{RF=100\%}$) and summarized in **Fig. S14F.** The oscillation frequency decreases as the OTFTs become more and more non-ideal, due to smaller carrier mobility values of non-ideal OTFTs around operating point thus larger propagation delay time(*70*) The oscillator based on OTFTs with 101% reliability factor has almost identical frequency to that of ideal OTFTs at all supply voltages, while the difference between non-ideal OFTFs and ideal OTFTs enlarges as the supply voltage decreases; for instance, $f_{RF=80\%}/f_{RF=100\%}$ equals to 82.5% at 50V and decreases to 45.2% at 15V. Notably, the oscillator based on OTFTs with 20% reliability factor has oscillation frequency two orders of magnitude smaller than ideal OTFTs at 50V and shows negligible frequency and amplitude when the supply voltage decreases below 30V. These results indicate that low reliability factor not only degrades

circuit performance but also blocks its normal operation completely under certain conditions, creating obstacles in designing functional circuits and predicting their performance in real applications.

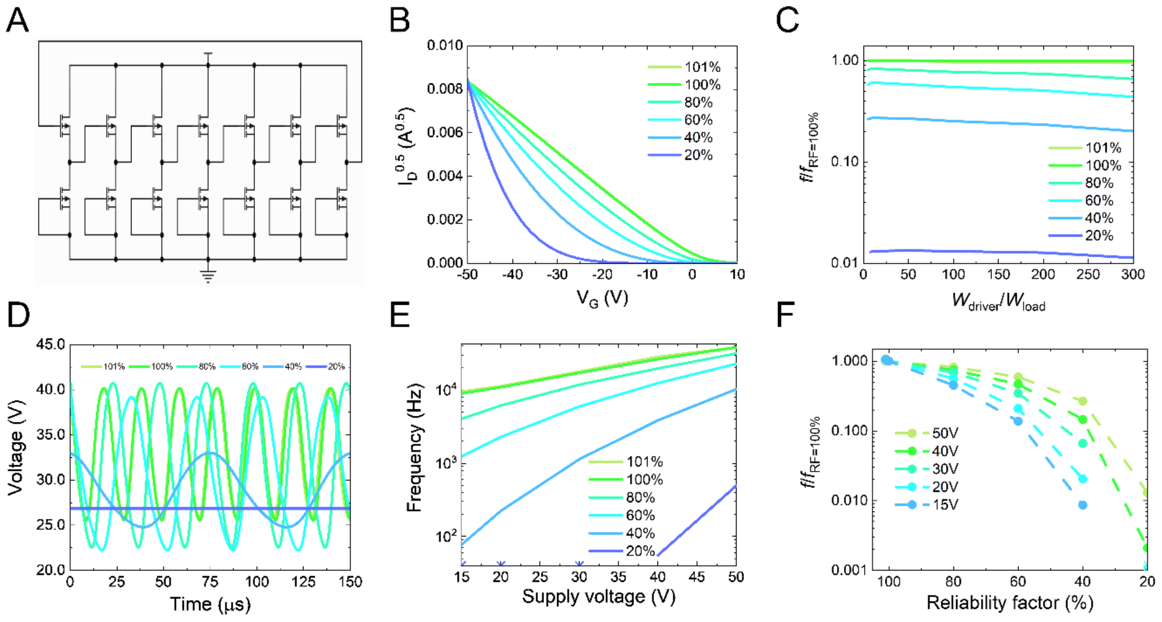

Fig. S14. Simulation results of seven-stage ring oscillators based on OTFTs.

(**A**) Circuit schematic of seven-stage ring oscillator. (**B**) Transfer curves of OTFTs with different reliability factors. (**C**) Oscillation frequencies of seven-stage ring oscillators relative to the frequency at 100% reliability factor versus W_{drive}/W_{load} at supply voltage of 50V. (**D**) Output waveform of oscillators with different reliability factor at supply voltage of 50V and W_{drive}/W_{load} of 5. (**E**) Oscillation frequency of oscillators with different reliability factors versus supply voltage at W_{driver}/W_{load} of 7.5; (**F**) Voltage dependent oscillation frequency of seven-stage ring oscillators relative to the frequency at 100% reliability factor versus the reliability factor of the constructing OTFTs.

REFERENCES AND NOTES

1. M. Zhu, Y. Guo, Y. Liu, A thriving decade: rational design, green synthesis, and cutting-edge applications of isoindigo-based conjugated polymers in organic field-effect transistors. *Sci. China Chem.* **65**, 1225–1264 (2022).

2. S. Wang, S. Fabiano, S. Himmelberger, S. Puzinas, X. Crispin, A. Salleo, M. Berggren, Experimental evidence that short-range intermolecular aggregation is sufficient for efficient charge transport in conjugated polymers. *Proc. Natl. Acad. Sci. U.S.A.* **112**, 10599–10604 (2015).

3. Y. Zhao, C. Su, G. Shen, Z. Xie, W. Xiao, Y. Fu, S. Inal, Q. Wang, Y. Wang, W. Yue, I. McCulloch, D. He, Donor Engineering Tuning the Analog Switching Range and Operational Stability of Organic Synaptic Transistors for Neuromorphic Systems. *Adv. Funct. Mater.* **32**, 2205744 (2022).

4. S. Chen, S. Zhu, Z. Lin, J. Peng, Transforming Polymorphs via Meniscus-Assisted Solution-Shearing Conjugated Polymers for Organic Field-Effect Transistors. *ACS Nano* **16**, 11194–11203 (2022).

5. C. S. Buga, J. C. Viana, A Review on Materials and Technologies for Organic Large-Area Electronics. *Adv. Mater. Technol.* **6**, 2001016 (2021).

6. M. Xiao, B. Kang, S. B. Lee, L. M. A. Perdigão, A. Luci, D. A. Warr, S. P. Senanayak, M. Nikolka, M. Statz, Y. Wu, A. Sadhanala, S. Schott, R. Carey, Q. Wang, M. Lee, C. Kim, A. Onwubiko, C. Jellett, H. Liao, W. Yue, K. Cho, G. Costantini, I. McCulloch, H. Sirringhaus, Anisotropy of Charge Transport in a Uniaxially Aligned Fused Electron-Deficient Polymer Processed by Solution Shear Coating. *Adv. Mater.* **32**, 2000063 (2020).

7. M. Xiao, A. Sadhanala, M. Abdi-Jalebi, T. H. Thomas, X. Ren, T. Zhang, H. Chen, R. L. Carey, Q. Wang, S. P. Senanayak, C. Jellett, A. Onwubiko, M. Moser, H. Liao, W. Yue, I. McCulloch, M. Nikolka, H. Sirringhaus, Linking Glass-Transition Behavior to Photophysical and Charge Transport Properties of High-Mobility Conjugated Polymers. *Adv. Funct. Mater.* **31**, 2007359 (2021).

8. J. Xu, S. Wang, G.-J. N. Wang, C. Zhu, S. Luo, L. Jin, X. Gu, S. Chen, V. R. Feig, J. W. F. To, S. Rondeau-Gagné, J. Park, B. C. Schroeder, C. Lu, J. Y. Oh, Y. Wang, Y.-H. Kim, H. Yan, R. Sinclair, D. Zhou, G. Xue, B. Murmann, C. Linder, W. Cai, J. B.-H. Tok, J. W. Chung, Z. Bao, Highly

stretchable polymer semiconductor films through the nanoconfinement effect. *Science* **355**, 59–64 (2017).

9. H. Liao, J. Chen, L. Lan, Y. Yu, G. Zhu, J. Duan, X. Zhu, H. Dai, M. Xiao, Z. Li, W. Yue, I. McCulloch, Efficient n-Type Small-Molecule Mixed Ion-Electron Conductors and Application in Hydrogen Peroxide Sensors. *ACS Appl. Mater. Interfaces* **14**, 16477–16486 (2022).

10. D. Ohayon, S. Inal, Organic Bioelectronics: From Functional Materials to Next-Generation Devices and Power Sources. *Adv. Mater.* **32**, 2001439 (2020).

11. K. Guo, S. Wustoni, A. Koklu, E. Díaz-Galicia, M. Moser, A. Hama, A. A. Alqahtani, A. N. Ahmad, F. S. Alhamlan, M. Shuaib, A. Pain, I. McCulloch, S. T. Arold, R. Grünberg, S. Inal, Rapid single-molecule detection of COVID-19 and MERS antigens via nanobody-functionalized organic electrochemical transistors. *Nat. Biomed. Eng.* **5**, 666–677 (2021).

12. G. D. Spyropoulos, J. N. Gelinas, D. Khodagholy, Internal ion-gated organic electrochemical transistor: A building block for integrated bioelectronics. *Sci. Adv.* **5**, eaau7378 (2019).

13. E. Macchia, K. Manoli, B. Holzer, C. Di Franco, M. Ghittorelli, F. Torricelli, D. Alberga, G. F. Mangiatordi, G. Palazzo, G. Scamarcio, L. Torsi, Single-molecule detection with a millimetre-sized transistor. *Nat. Commun.* **9**, 3223 (2018).

14. S. Wang, M. Kappl, I. Liebewirth, M. Müller, K. Kirchhoff, W. Pisula, K. Müllen, Organic Field-Effect Transistors based on Highly Ordered Single Polymer Fibers. *Adv. Mater.* **24**, 417–420 (2012).

15. H. H. Choi, K. Cho, C. D. Frisbie, H. Sirringhaus, V. Podzorov, Critical assessment of charge mobility extraction in FETs. *Nat. Mater.* **17**, 2 (2018), 7.

16. D. Venkateshvaran, M. Nikolka, A. Sadhanala, V. Lemaur, M. Zelazny, M. Kepa, M. Hurhangee, A. J. Kronemeijer, V. Pecunia, I. Nasrallah, I. Romanov, K. Broch, I. McCulloch, D. Emin, Y. Olivier, J. Cornil, D. Beljonne, H. Sirringhaus, Approaching disorder-free transport in high-mobility conjugated polymers. *Nature* **515**, 384–388 (2014).

17. J. Wang, Y. Liu, L. Hua, T. Wang, H. Dong, H. Li, X. Sun, Z. Ren, S. Yan, Oriented Conjugated Copolymer Films with Controlled Crystal Forms and Molecular Stacking Modes for Enhanced Charge Transport and Photoresponsivity. *ACS Appl. Polym. Mater.* **3**, 2098–2108 (2021).

18. Z. Wang, Z. Liu, L. Ning, M. Xiao, Y. Yi, Z. Cai, A. Sadhanala, G. Zhang, W. Chen, H. Sirringhaus, D. Zhang, Charge Mobility Enhancement for Conjugated DPP-Selenophene Polymer by Simply Replacing One Bulky Branching Alkyl Chain with Linear One at Each DPP Unit. *Chem. Mater.* **30**, 3090–3100 (2018).

19. W. Yue, M. Nikolka, M. Xiao, A. Sadhanala, C. B. Nielsen, A. J. P. White, H.-Y. Chen, A. Onwubiko, H. Sirringhaus, I. McCulloch, Azaisoindigo conjugated polymers for high performance n-type and ambipolar thin film transistor applications. *J. Mater. Chem. C* **4**, 9704–9710 (2016).

20. S. Fratini, M. Nikolka, A. Salleo, G. Schweicher, H. Sirringhaus, Charge transport in high-mobility conjugated polymers and molecular semiconductors. *Nat. Mater.* **19**, 491–502 (2020).

21. S. D. Ogier, J. Veres, D. C. Cupertino, S. Mohialdin Khaffaf, S. W. Leeming, Low-*k* Insulators as the Choice of Dielectrics in Organic Field-Effect Transistors. *Adv. Funct. Mater.* **13**, 199–204 (2003).

22. M. Fahlman, S. Fabiano, V. Gueskine, D. Simon, M. Berggren, X. Crispin, Interfaces in organic electronics. *Nat. Rev. Mater.* **4**, 627–650 (2019).

23. M. Nikolka, I. Nasrallah, B. Rose, M. K. Ravva, K. Broch, A. Sadhanala, D. Harkin, J. Charmet, M. Hurhangee, A. Brown, S. Illig, P. Too, J. Jongman, I. McCulloch, J.-L. Bredas, H. Sirringhaus, High operational and environmental stability of high-mobility conjugated polymer field-effect transistors through the use of molecular additives. *Nat. Mater.* **16**, 356–362 (2017).

24. A. Nedungadi, T. Viswanathan, Design of linear CMOS transconductance elements. *IEEE Trans. Circuits Syst.* **31**, 891–894 (1984).

25. R. van Langevelde, F. M. Klaassen, Effect of gate-field dependent mobility degradation on distortion analysis in MOSFETs. *IEEE Trans. Electron Devices* **44**, 2044–2052 (1997).

26. W. Zhang, J. Smith, S. E. Watkins, R. Gysel, M. McGehee, A. Salleo, J. Kirkpatrick, S. Ashraf, T. Anthopoulos, M. Heeney, I. McCulloch, Indacenodithiophene Semiconducting Polymers for High-Performance, Air-Stable Transistors. *J. Am. Chem. Soc.* **132**, 11437–11439 (2010).

27. H. Yan, Z. Chen, Y. Zheng, C. Newman, J. R. Quinn, F. Dötz, M. Kastler, A. Facchetti, A high-mobility electron-transporting polymer for printed transistors. *Nature* **457**, 679–686 (2009).

28. D. Simatos, L. J. Spalek, U. Kraft, M. Nikolka, X. Jiao, C. R. McNeill, D. Venkateshvaran, H. Sirringhaus, The effect of the dielectric end groups on the positive bias stress stability of N2200 organic field effect transistors. *APL Mater.* **9**, 041113 (2021).

29. R. Di Pietro, I. Nasrallah, J. Carpenter, E. Gann, L. S. Kölln, L. Thomsen, D. Venkateshvaran, K. O'Hara, A. Sadhanala, M. Chabinyc, C. R. McNeill, A. Facchetti, H. Ade, H. Sirringhaus, D. Neher, Coulomb Enhanced Charge Transport in Semicrystalline Polymer Semiconductors. *Adv. Funct. Mater.* **26**, 8011–8022 (2016).

30. W. B. Jackson, N. M. Amer, A. C. Boccara, D. Fournier, Photothermal deflection spectroscopy and detection. *Appl. Optics* **20**, 1333–1344 (1981).

31. W. B. Jackson, N. M. Amer, Direct measurement of gap-state absorption in hydrogenated amorphous silicon by photothermal deflection spectroscopy. *Phys. Rev. B* **25**, 5559–5562 (1982).

32. T. H. Thomas, D. J. Harkin, A. J. Gillett, V. Lemaur, M. Nikolka, A. Sadhanala, J. M. Richter, J. Armitage, H. Chen, I. McCulloch, S. M. Menke, Y. Olivier, D. Beljonne, H. Sirringhaus, Short contacts between chains enhancing luminescence quantum yields and carrier mobilities in conjugated copolymers. *Nat. Commun.* **10**, 2614 (2019).

33. T. H. Thomas, J. P. H. Rivett, Q. Gu, D. J. Harkin, J. M. Richter, A. Sadhanala, C. K. Yong, S. Schott, K. Broch, J. Armitage, A. J. Gillett, S. M. Menke, A. Rao, D. Credgington, H. Sirringhaus, Chain Coupling and Luminescence in High-Mobility, Low-Disorder Conjugated Polymers. *ACS Nano.* **13**, 13716–13727 (2019).

34. M. Li, D. K. Mangalore, J. Zhao, J. H. Carpenter, H. Yan, H. Ade, H. Yan, K. Müllen, P. W. M. Blom, W. Pisula, D. M. de Leeuw, K. Asadi, Integrated circuits based on conjugated polymer monolayer. *Nat. Commun.* **9**, 451 (2018).

35. M. Caironi, M. Bird, D. Fazzi, Z. Chen, R. Di Pietro, C. Newman, A. Facchetti, H. Sirringhaus, Very Low Degree of Energetic Disorder as the Origin of High Mobility in an n-channel Polymer Semiconductor. *Adv. Funct. Mater.* **21**, 3371–3381 (2011).

36. A. J. Kronemeijer, V. Pecunia, D. Venkateshvaran, M. Nikolka, A. Sadhanala, J. Moriarty, M. Szumilo, H. Sirringhaus, Two-Dimensional Carrier Distribution in Top-Gate Polymer Field-Effect Transistors: Correlation between Width of Density of Localized States and Urbach Energy. *Adv. Mater.* **26**, 728–733 (2014).

37. Y. Olivier, D. Niedzialek, V. Lemaur, W. Pisula, K. Müllen, U. Koldemir, J. R. Reynolds, R. Lazzaroni, J. Cornil, D. Beljonne, 25th Anniversary Article: High-Mobility Hole and Electron Transport Conjugated Polymers: How Structure Defines Function. *Adv. Mater.* **26**, 2119–2136 (2014).

38. Y. Liu, J. Zhao, Z. Li, C. Mu, W. Ma, H. Hu, K. Jiang, H. Lin, H. Ade, H. Yan, Aggregation and morphology control enables multiple cases of high-efficiency polymer solar cells. *Nat. Commun.* **5**, 5293 (2014).

39. V. Dantanarayana, J. Fuzell, D. Nai, I. E. Jacobs, H. Yan, R. Faller, D. Larsen, A. J. Moule, Put Your Backbone into It: Excited-State Structural Relaxation of PffBT4T-2DT Conducting Polymer in Solution. *J. Phys. Chem. C* **122**, 7020–7026 (2018).

40. J. Zhao, Y. Li, H. Lin, Y. Liu, K. Jiang, C. Mu, T. Ma, J. Y. Lin Lai, H. Hu, D. Yu, H. Yan, High-efficiency non-fullerene organic solar cells enabled by a difluorobenzothiadiazole-based donor polymer combined with a properly matched small molecule acceptor. *Energ. Environ. Sci.* **8**, 520–525 (2015).

41. D. Baran, T. Kirchartz, S. Wheeler, S. Dimitrov, M. Abdelsamie, J. Gorman, R. S. Ashraf, S. Holliday, A. Wadsworth, N. Gasparini, P. Kaienburg, H. Yan, A. Amassian, C. J. Brabec, J. R.

Durrant, I. McCulloch, Reduced voltage losses yield 10% efficient fullerene free organic solar cells with >1 V open circuit voltages. *Energ. Environ. Sci.* **9**, 3783–3793 (2016).

42. V. Nádaždy, F. Schauer, K. Gmucová, Energy resolved electrochemical impedance spectroscopy for electronic structure mapping in organic semiconductors. *Appl. Phys. Lett.* **105**, 142109 (2014).

43. I. McCulloch, M. Heeney, C. Bailey, K. Genevicius, I. MacDonald, M. Shkunov, D. Sparrowe, S. Tierney, R. Wagner, W. Zhang, M. L. Chabinyc, R. J. Kline, M. D. McGehee, M. F. Toney, Liquid-crystalline semiconducting polymers with high charge-carrier mobility. *Nat. Mater.* **5**, 328–333 (2006).

44. C. Cendra, L. Balhorn, W. Zhang, K. O'Hara, K. Bruening, C. J. Tassone, H.-G. Steinrück, M. Liang, M. F. Toney, I. McCulloch, M. L. Chabinyc, A. Salleo, C. J. Takacs, Unraveling the Unconventional Order of a High-Mobility Indacenodithiophene–Benzothiadiazole Copolymer. *ACS Macro Lett.* **10**, 1306–1314 (2021).

45. T. Schuettfort, B. Watts, L. Thomsen, M. Lee, H. Sirringhaus, C. R. McNeill, Microstructure of Polycrystalline PBTTT Films: Domain Mapping and Structure Formation. *ACS Nano* **6**, 1849–1864 (2012).

46. D. Moerman, G. E. Eperon, J. T. Precht, D. S. Ginger, Correlating Photoluminescence Heterogeneity with Local Electronic Properties in Methylammonium Lead Tribromide Perovskite Thin Films. *Chem. Mater.* **29**, 5484–5492 (2017).

47. M. M. Nahid, E. Gann, L. Thomsen, C. R. McNeill, NEXAFS spectroscopy of conjugated polymers. *Eur. Polym. J.* **81**, 532–554 (2016).

48. R. Noriega, J. Rivnay, K. Vandewal, F. P. V. Koch, N. Stingelin, P. Smith, M. F. Toney, A. Salleo, A general relationship between disorder, aggregation and charge transport in conjugated polymers. *Nat. Mater.* **12**, 1038–1044 (2013).

49. I. E. Jacobs, G. D'Avino, V. Lemaur, Y. Lin, Y. Huang, C. Chen, T. F. Harrelson, W. Wood, L. J. Spalek, T. Mustafa, C. A. O'Keefe, X. Ren, D. Simatos, D. Tjhe, M. Statz, J. W. Strzalka, J.-K. Lee, I. McCulloch, S. Fratini, D. Beljonne, H. Sirringhaus, Structural and Dynamic Disorder, Not Ionic

Trapping, Controls Charge Transport in Highly Doped Conducting Polymers. *J. Am. Chem. Soc.* **144**, 3005–3019 (2022).

50. M. Statz, D. Venkateshvaran, X. Jiao, S. Schott, C. R. McNeill, D. Emin, H. Sirringhaus, R. Di Pietro, On the manifestation of electron-electron interactions in the thermoelectric response of semicrystalline conjugated polymers with low energetic disorder. *Commun. Phys.* **1**, 16 (2018).

51. X. Zhang, H. Bronstein, A. J. Kronemeijer, J. Smith, Y. Kim, R. J. Kline, L. J. Richter, T. D. Anthopoulos, H. Sirringhaus, K. Song, M. Heeney, W. Zhang, I. McCulloch, D. M. DeLongchamp, Molecular origin of high field-effect mobility in an indacenodithiophene–benzothiadiazole copolymer. *Nat. Commun.* **4**, 2238 (2013).

52. R. J. Kline, M. D. McGehee, E. N. Kadnikova, J. Liu, J. M. J. Fréchet, M. F. Toney, Dependence of Regioregular Poly(3-hexylthiophene) Film Morphology and Field-Effect Mobility on Molecular Weight. *Macromolecules* **38**, 3312–3319 (2005).

53. X. Zhang, S. D. Hudson, D. M. DeLongchamp, D. J. Gundlach, M. Heeney, I. McCulloch, In-Plane Liquid Crystalline Texture of High-Performance Thienothiophene Copolymer Thin Films. *Adv. Funct. Mater.* **20**, 4098–4106 (2010).

54. H. Sirringhaus, P. J. Brown, R. H. Friend, M. M. Nielsen, K. Bechgaard, B. M. W. Langeveld-Voss, A. J. H. Spiering, R. A. J. Janssen, E. W. Meijer, P. Herwig, D. M. de Leeuw, Two-dimensional charge transport in self-organized, high-mobility conjugated polymers. *Nature* **401**, 685–688 (1999).

55. D. Venkateshvaran, A. J. Kronemeijer, J. Moriarty, D. Emin, H. Sirringhaus, Field-effect modulated Seebeck coefficient measurements in an organic polymer using a microfabricated on-chip architecture. *APL Mater.* **2**, 032102 (2014).

56. J. Shamsi, D. Kubicki, M. Anaya, Y. Liu, K. Ji, K. Frohna, C. P. Grey, R. H. Friend, S. D. Stranks, Stable Hexylphosphonate-Capped Blue-Emitting Quantum-Confined $CsPbBr_3$ Nanoplatelets. *ACS Energy Lett.* **5**, 1900–1907 (2020).

57. M. Xiao, R. L. Carey, H. Chen, X. Jiao, V. Lemaur, S. Schott, M. Nikolka, C. Jellett, A. Sadhanala, S. Rogers, S. P. Senanayak, A. Onwubiko, S. Han, Z. Zhang, M. Abdi-Jalebi, Y. Zhang, T. H.

Thomas, N. Mahmoudi, L. Lai, E. Selezneva, X. Ren, M. Nguyen, Q. Wang, I. Jacobs, W. Yue, C. R. McNeill, G. Liu, D. Beljonne, I. McCulloch, H. Sirringhaus, Charge transport physics of a unique class of rigid-rod conjugated polymers with fused-ring conjugated units linked by double carbon-carbon bonds. *Sci. Adv.* **7**, eabe5280 (2021).

58. J. Liu, S. Chen, D. Qian, B. Gautam, G. Yang, J. Zhao, J. Bergqvist, F. Zhang, W. Ma, H. Ade, O. Inganäs, K. Gundogdu, F. Gao, H. Yan, Fast charge separation in a non-fullerene organic solar cell with a small driving force. *Nat. Energy* **1**, 16089 (2016).

59. F. C. Spano, The spectral signatures of frenkel polarons in H- And J-aggregates. *Acc. Chem. Res.* **43**, 429–439 (2010).

60. A. C. Arias, J. D. MacKenzie, I. McCulloch, J. Rivnay, A. Salleo, Materials and Applications for Large Area Electronics: Solution-Based Approaches. *Chem. Rev.* **110**, 3–24 (2010).

61. Z. Yao, Y. Zheng, J. Dou, Y. Lu, Y. Ding, L. Ding, J. Wang, J. Pei, Approaching Crystal Structure and High Electron Mobility in Conjugated Polymer Crystals. *Adv. Mater.* **33**, 2006794 (2021).

62. Z. Yao, Q. Li, H. Wu, Y. Ding, Z. Wang, Y. Lu, J. Wang, J. Pei, Building crystal structures of conjugated polymers through X-ray diffraction and molecular modeling. *SmartMat* **2**, 378–387 (2021).

63. S. L. Mayo, B. D. Olafson, W. A. Goddard, DREIDING: A generic force field for molecular simulations. *J. Phys. Chem.* **94**, 8897–8909 (1990).

64. J. Gasteiger, M. Marsili, Iterative partial equalization of orbital electronegativity—A rapid access to atomic charges. *Tetrahedron* **36**, 3219–3228 (1980).

65. J.-F. Chang, M. C. Gwinner, M. Caironi, T. Sakanoue, H. Sirringhaus, Conjugated-Polymer-Based Lateral Heterostructures Defined by High-Resolution Photolithography. *Adv. Funct. Mater.* **20**, 2825–2832 (2010).

66. D. Emin, *Polarons* (Cambridge Univ. Press, 2012).

67. D. Emin, Enhanced Seebeck coefficient from carrier-induced vibrational softening. *Phys. Rev. B* **59**, 6205–6210 (1999).

68. G. M. Heric, A. F. Tasch, S. K. Banerjee, A universal MOSFET mobility degradation model for circuit simulation. *IEEE Trans. Comput. Aided Des. Integr. Circuits Syst.* **9**, 1123–1126 (1990).

69. F. Babarada, M. D. Profirescu, A. Rusu, in *2003 International Semiconductor Conference. CAS 2003 Proceedings (IEEE Cat. No.03TH8676)* (IEEE Operations Center, 2003); http://ieeexplore.ieee.org/document/1252440/, pp. 301–304.

70. K. Lee, J. Jang, S. Choi, D. Kim, K. Kim, D. Kim, Extraction of Propagation Delay-Correlated Mobility and Its Verification for Amorphous InGaZnO Thin-Film Transistor-Based Inverters. *IEEE Trans. Electron Devices* **62**, 1504–1510 (2015).
